# Rapeseed Oil as Feedstock for Bio-Based Thermoset Foams Obtained via Michael Addition Reaction

**DOI:** 10.3390/polym16010117

**Published:** 2023-12-29

**Authors:** Mikelis Kirpluks, Arnis Abolins, Darta Eihe, Ralfs Pomilovskis, Anda Fridrihsone

**Affiliations:** 1 Polymer Laboratory, Latvian State Institute of Wood Chemistry, Str. Dzerbenes 27, LV-1006 Riga, Latvia; arnis.abolins@kki.lv (A.A.); darta.eihe@kki.lv (D.E.); ralfs.pomilovskis@kki.lv (R.P.); anda.fridrihsone@kki.lv (A.F.); 2 Institute of Technology of Organic Chemistry, Faculty of Materials Science and Applied Chemistry, Riga Technical University, Str. P. Valdena 3/7, LV-1048 Riga, Latvia

**Keywords:** rapeseed oil, Michael donors, polymer foams, thermal insulation

## Abstract

Rapeseed oil was used to develop thermoset foams via Michael addition reaction by mixing two liquid components, Michael donor and Michael acceptor. The foaming of the curing thermoset was achieved by the physical blowing agent which expanded from the reacting foam mass due to an exothermic curing reaction. The influence of the rapeseed oil-based Michael donor functionality on the foaming process and the characteristics of the obtained thermoset foams was studied. The 1,1,3,3-tetramethylguanidine catalyst’s influence on the foaming process kinetics was studied using FOAMAT equipment. The curing of the bio-based thermoset was analysed using a dielectric polarisation sensor. The morphology of the developed thermoset foam was analysed using a scanning electron microscope and the obtained foams were characterized using TGA, DSC, DMA and mechanical analysis tests. A direct correlation between the thermoset foam polymer crosslinking density and foaming reactivity, mechanical properties and glass transition temperature were determined. Obtained rapeseed oil based thermoset foams had a relatively low thermal conductivity of 33.9–35.4 mW/(m·K) which allows their use as thermal insulation material in civil engineering applications.

## 1. Introduction

The bio-economy plays a pivotal role in addressing some of our planet’s most pressing challenges. The bio-economy offers a sustainable and viable alternative by harnessing the power of biological resources such as plants [1] and microorganisms [2,3,4] to produce energy [2,5,6], materials [7] and other products [8]. Moreover, the bio-economy fosters innovation and creates new economic opportunities across various sectors, leading to job creation and economic growth [8]. By embracing the bio-economy, we can pave the way towards a greener, more resilient and prosperous future while safeguarding biodiversity and preserving the delicate balance of our ecosystems for generations to come.

Thermal insulation is crucial for energy efficiency due to its ability to minimize heat transfer between the interior and exterior of a building or structure [9]. It acts as a barrier, reducing the amount of heat that enters or leaves a space, which is crucial for maintaining comfortable indoor temperatures. By providing effective thermal insulation, buildings can significantly reduce their reliance on heating and cooling systems, resulting in lower energy consumption [10]. As energy consumption decreases due to improved thermal insulation, there is a reduction in the burning of fossil fuels to generate electricity, leading to lower greenhouse gas emissions and a positive impact on climate change [11]. Energy-efficient buildings with proper thermal insulation align with sustainability goals by reducing their ecological footprint and minimizing resource consumption [12]. Thermal insulation is a crucial component of energy-efficient buildings and structures. As the world moves towards a more sustainable future, promoting energy efficiency through thermal insulation becomes increasingly important in mitigating the impact of human activities on the environment [13].

Civil engineering utilizes a variety of thermal insulation materials to enhance energy efficiency and provide comfort in buildings and infrastructure. The choice of thermal insulation material depends on various factors, including the building’s design, climate conditions, budget and environmental considerations [14]. 

Fibreglass and mineral wool are one of the most widely used thermal insulation materials. It consists of fine glass/mineral fibres bound together with a resin. It is available in various forms, such as rolls, batts and loose fill [15]. Fibreglass and mineral wool insulation are cost-effective, non-combustible and easy to install. However, their thermal conductivity is 30.0–45.0 mW/(m∙K) [16], which is a bit higher than for closed-cell rigid polyurethane (PU) foam. Furthermore, glass/mineral fibre insulation has a potential problem with water or moisture absorption as the material has an open cell structure. This could lead to the growth of fungi in building walls, resulting in a decrease in living space comfort and a decrease in the thermal insulation properties [17]. 

Expanded polystyrene (EPS) and extruded polystyrene (XPS) are rigid foam boards commonly used in building construction. They have good thermal resistance, are moisture-resistant and are often used in insulating walls, roofs and foundations (31.0–38.0 mW/(m·K)) [18]. The decreased flammability of EPS and XPS is achieved by adding various flame retardants, which increase the final product’s price and may cause health concerns [19].

Vacuum insulation panels (VIPs) and aerogel insulation are high-performance insulation panels that use a vacuum to minimize heat transfer [20]. They have a very low thermal conductivity of 3.5–8.0 mW/(m·K) in the case of VIPs [21] and 14.0–23.0 mW/(m·K) in the case of aerogels [22] and are suitable for applications where space is limited. Despite their excellent thermal insulation properties, VIP and aerogel insulation application is limited due to extremely high material costs, which are several times higher than other alternatives [23]. 

One of the best thermal insulation materials is rigid PU foam, which provides excellent thermal insulation as its thermal conductivity is 20.0–30.0 mW/(m·K) [24]. However, it has several significant drawbacks which will limit its application in the post-fossil economy. One of the drawbacks of rigid PU foam is its potential contribution to environmental pollution. While rigid PU foam is widely used for its excellent insulation properties and structural support, it has some environmental concerns, such as ozone depletion and global warming potential [25]. Some rigid PU foam products may contain blowing agents, such as hydrochlorofluorocarbons (HCFCs) or hydrofluorocarbons (HFCs), which are potent greenhouse gases and contribute to ozone depletion when released into the atmosphere [26]. The blowing agents used in rigid PU foam can have high global warming potential, meaning they can trap heat in the atmosphere and contribute to climate change [27].

Furthermore, rigid PU foam is not easily biodegradable, posing challenges for waste disposal and recycling. Improper disposal or incineration can lead to the release of toxic emissions and landfill waste accumulation [28]. The production of rigid PU foam typically relies on petroleum-based feedstocks, which are derived from non-renewable resources and contribute to the depletion of finite fossil fuels. The bio-based polyurethane market share in 2023 was only 8.98% [29]. It has been shown that only one of the rigid PU foam components can feasibly be produced economically from bio-based feedstocks – bio-based polyols [30,31,32,33]. The other component—polyisocyanate—is a petrochemical product, and it is unlikely that it will be produced in a sustainable manner [34,35,36]. 

To address these environmental drawbacks, manufacturers and researchers are actively exploring more sustainable alternatives for rigid PU foam. This includes developing blowing agents with lower global warming potential [37], incorporating bio-based or recycled content into foams [38] and improving recycling and waste management strategies for end-of-life products [39]. Additionally, regulatory measures and certifications encourage the use of eco-friendly materials and practices to minimize the environmental impact of rigid PU foam [40]. However, several health concerns are still related to rigid PU foam thermal insulation. During the production and installation of rigid PU foam, certain chemical components, such as isocyanates, can be hazardous to human health if proper safety measures are not followed [41]. Exposure to isocyanates can cause skin and respiratory sensitization, leading to health risks for workers and occupants [42]. Thus, it is vital to find an alternative to rigid PU foam thermal insulation. 

In this article, we propose an alternative method to rigid PU foams by developing rigid thermoset foams from bio-based feedstock using similar production technology as the rigid thermoset foams were obtained by mixing two different liquid components. The principle thermoset polymerization reaction was carbon Michael addition between polyfunctional acetoacetate (Michael donor) and polyfunctional acrylate (Michael acceptor). The curing reaction was carried out at room temperature and the foaming of the material was achieved by physical blowing agents. An industrial crop product, rapeseed oil (RO), was used to synthesize Michael’s donors with varied reacting group functionality, and the Michael donor functionality influence on the foamed polymer properties was studied. The proposed technology allows one to obtain rigid polymeric foams with similar properties to rigid PU foams while bypassing the main rigid PU foam drawback—the use of isocyanates.

## 2. Materials and Methods

### 2.1. Materials for Michael Foam Production

Epoxidized rapeseed oil (ERO) polyol acetoacetates were obtained from polyols that were synthesized from rapeseed oil via epoxidation using ion exchange resin followed by oxirane ring-opening and esterification with methanol, 1,4-butanediol, diethylene glycol and trimethylolpropane and subsequent acetoacetylation with *tert*-butyl acetoacetate by a transesterification reaction. The ERO-basedacetoacetate ERO_MeOH_AA was obtained from ERO by oxirane ring-opening and esterification with methanol and subsequent acetoacetylation with *tert*-butyl acetoacetate by a transesterification reaction, acid value < 5 mg KOH·g^−1^, hydroxyl value 25 mg KOH·g^−1^, apparent viscosity 6300 mPa∙s (y = 50 s^−1^), and acetoacetate groups 0.2410 mol·100 g^−1^. The acetoacetate ERO_BD_AA was obtained from ERO by oxirane ring-opening and esterification with 1,4-butanediol and subsequent acetoacetylation with *tert*-butyl acetoacetate by a transesterification reaction, acid value < 5 mg KOH·g^−1^, hydroxyl value 50 mg KOH·g^−1^, apparent viscosity 5265 mPa∙s (y = 50 s^−1^), and acetoacetate groups 0.3908 mol·100 g^−1^. The acetoacetate ERO_DEG_AA was obtained from ERO by oxirane ring-opening and esterification with diethylene glycol and subsequent acetoacetylation with *tert*-butyl acetoacetate by a transesterification reaction, acid value < 5 mg KOH·g^−1^, hydroxyl value 50 mg KOH·g^−1^, apparent viscosity 3780 mPa∙s (y = 50 s^−1^) and acetoacetate groups 0.3905 mol·100 g^−1^. The acetoacetate ERO_TMP_AA was obtained from ERO by oxirane ring-opening and esterification with methanol and subsequent acetoacetylation with *tert*-butyl acetoacetate by a transesterification reaction, acid value < 5 mg KOH·g^−1^, hydroxyl value 60 mg KOH·g^−1^, apparent viscosity 25,185 mPa∙s (y = 50 s^−1^) and acetoacetate groups 0.4346 mol·100 g^−1^. All of the used ERO-polyol acetoacetates were synthesized at the Latvian State Institute of Wood Chemistry.

Trimethylolpropanetriacrylate (TMPTA) contained 600 ppm monomethyl ether hydroquinone as an inhibitor and is of technical grade from Sigma-Aldrich, Schnelldorf, Germany. 1,1,3,3-tetramethylguanidine (TMG), assay 99%, was purchased from Sigma-Aldrich. Tegostab B 8870 (TG-B) was used as a surfactant and was purchased from Evonik Industries, Essen, Germany. Solstice^®^ LBA was used as a physical blowing agent and was purchased from Honeywell, Charlotte, the United States. Acrylate, catalyst, surfactant, and blowing agent were used directly as delivered.

### 2.2. Screening of Catalyst Amount Influence for Obtaining Michael Foam

The Michael foams were prepared, according to the formulations reported in Table 1, by mixing the Michael donor and Michael acceptor components. The ratio of acetoacetate to acrylic groups was 1:2 mol. The catalyst was used as 2%, 3%, 4%, 5% and 6% of Michael’s donor mass. At first, acrylate, catalyst, surfactant, and blowing agent components were weighed and mixed in a 50 mL closed plastic cup using a planetary centrifuge for 25 s at 2500 rpm to thoroughly mix and ensure homogeneity of the prepared medium. A very small part of the blowing agent evaporated during the first mixing, so the lost amount was compensated. After that, the Michael donor was added, and all components were mixed again for another 25 s at 2500 rpm. The obtained medium occurred homogenous. The plastic lid was removed to allow the foam to free-rise in a plastic cup. The height of the raised foam was registered using an ultrasonic sensor of Foam Qualification System FOAMAT^®^ 285, Format Messtechnik GmbH, Karlsruhe, Germany.

The following acronyms were used for each obtained Michael foam: MF_MeOH, MF_BD, MF_DEG, and MF_TMP.

### 2.3. Determination of Dielectric Polarization

To investigate the reaction between four different functionality acetoacetates and TMPTA, a series of experiments were conducted using TMG as a catalyst. The reaction was carried out by initially combining the catalyst and TMPTA, followed by adding an acetoacetate. The mixture was thoroughly mixed for 20 s using a planetary centrifuge mixer to ensure homogeneity. Subsequently, the resulting mixture was poured onto a Curing Monitor Device (CMD) sensor, allowing for the determination of dielectric polarization change over experiment time. This experimental approach aimed to assess and compare curing kinetics using dielectric polarization properties of the polymeric materials formed from the various acetoacetate functionalities in combination with TMPTA (MF_MeOH, MF_BD, MF_DEG, MF_TMP). To determine the change of dielectric polarization during polymer curing, a CMD sensor of Foam Qualification System FOAMAT^®^ 285 was used. The reaction mixture was poured onto the CMD-sensor, allowing for the determination of dielectric polarization and providing valuable insights into the electrochemical properties of samples during the transition from fluid to solid. The required number of reagents used in the reaction is presented in Table 2. The molar ratio of the utilized different functionality acetoacetates and TMPTA was 1:2. The amount of TMG catalyst used in the reaction was 3% of the mass of the acetoacetate. The total mass of the reaction mixture used was 10 g. 

### 2.4. Michael Foam Preparation and Formulation for Steel Mould

The Michael foams were prepared, according to the formulations reported in Table 1, by mixing the Michael donor and Michael acceptor components. The ratio of acetoacetate to acrylic groups was 1:2 mol. At first, acrylate, catalyst, surfactant, and blowing agent components were weighed and mixed in a plastic cup using a mechanical stirrer for 30 s at 2500 rpm until a homogeneous medium was obtained. Some part of the physical blowing agent evaporated during the first mixing, so the lost amount was compensated. Immediately after that, the Michael donor was added, and all components were remixed for another 30 s at 2500 rpm. The obtained medium occurred homogenously and was poured instantaneously into previously heated (40 °C) closed-type steel mould which was immediately covered with a steel lid with tiny holes for the foam’s degassing. After that, the closed steel mould was inserted into the preheated oven for 90 min at 40 °C. Finally, after 90 min of the curing process in the oven at 40 °C, the prepared Michael foam samples were taken out from the steel mould and allowed to continue to cure at ambient conditions for 24 h before any further tests. The obtained Michael foam samples were cut with a band saw for various tests. The foam quality was visually assessed, and images of the foam samples were captured by a camera. 

### 2.5. Foaming Parameters

The foaming parameters, such as start and end times, were determined with an ultrasonic sensor using the Foam Qualification System FOAMAT^®^ 285.

### 2.6. Apparent Density

The apparent density of the obtained foam samples was tested according to the ISO 845:2006 standard [43].

### 2.7. Thermal Conductivity

The thermal conductivity coefficient (λ) was tested with a FOX 200 by TA instruments-Water LLC, New Castle, DE, USA, according to the ISO 8301:1991 standard [44], at an average temperature of 10 °C (cold plate: 0 °C, and hot plate: +20 °C, sample dimensions: 200 × 200 × 30 mm). 

### 2.8. Fourier-Transform Infrared Spectroscopy (FTIR)

The chemical structure of the obtained foam was analysed using FTIR data, which was determined with a Thermo Scientific Nicolet iS50 spectrometer Thermo Fisher Scientific at a resolution of 4 cm^−1^ (32 scans). FTIR data were collected using the attenuated total reflectance technique with diamond crystals. A sample of the obtained foam was pressed against the prism and analysed.

### 2.9. Closed Cell Content

The closed cell content was measured and calculated, according to ISO 4590:2016 [45], with a pycnometer AccuPyc II 1340 for specimens with dimensions 30 × 30 × 55 mm. 

### 2.10. Compressive Strength and Modulus

The compressive strength and modulus parallel and perpendicular to the foam rise direction were tested, according to the requirements of the ISO 844:2021 standard [46], on a testing machine Zwick/Roell Z100 (maximum load-cell capacity 1 kN, the deformation rate: 10%/min) for cylinder specimens with a diameter and height of ~20 mm. These cylindrical samples were cut with a drill press using a crown drill bit. Six specimens were analysed for each foam sample, and the average value was taken along with the standard deviation.

### 2.11. Differential Scanning Calorimetry (DSC)

DSC was performed on a TA Instrument DSC Q1000 (TA Instruments, New Castle, DE, USA) under a nitrogen atmosphere, using approximately 5 mg of each grounded sample. The glass transition temperature T_g_ was determined by software as the midpoint temperature, corresponding to half of the heat flow difference between the extrapolated onset and the extrapolated end temperature (half-step method). The samples were initially heated from ambient temperature to 180 °C at a heating rate of 10 °C/min, cooling until −100 °C and, then heated again at a rate of 10 °C/min to 180 °C. The preheating step (from ambient temperature to 180 °C) was conducted to remove the non-reversible thermal effects. A second scan was used to determine the glass transition temperature.

### 2.12. Dynamical Mechanical Analysis (DMA)

The DMA was carried out with a Mettler Toledo DMA/SDTA861e (Mettler Toledo, Greifensee, Switzerland) with the following parameters: a temperature ranges from −60 °C to 180 °C, a ramp rate of 3 °C/min, a frequency of 1 Hz, an amplitude of 5 μm, and a maximal force of 1 N. The compression oscillation mode was used. Foam samples with a diameter of ~13 mm and a height of ~7 mm were used for the tests.

### 2.13. Thermogravimetry Analysis (TGA)

The samples were analysed using a Discovery TGA thermogravimetric analyser and autosampler by TA Instruments, New Castle, DE, USA). The foam samples were placed on platinum scale pans and heated in a nitrogen atmosphere at 10 °C/min in a temperature range between 30–700 °C. Three parallel samples of foam samples were tested and analysed. The data were processed using the TA Instruments TRIOS #5.0.0.44608 software and OriginPro 2021 9.8.0.200.

## 3. Results and Discussion

This study proposes a new method of producing bio-based thermoset polymer foams that may be applied as thermal insulation. The process is similar to rigid PU foam production technology, as the polymer foams are obtained by mixing two reacting components. Moreover, the foamed morphology is obtained due to the expansion of the physical blowing agent as the temperature inside the foaming polymer mass rises as a result of the exothermic curing reaction. The foaming process rate was optimized with the addition of different amounts of a catalyst, which allowed to design of the curing characteristics of the foamed material. The main benefit of the proposed approach is that it is possible to obtain a foamed thermoset with increased bio-based material content compared to commonly used rigid PU foams which have about 15–25% of bio-based material content [47]. Furthermore, the proposed process allows to bypass the use of harmful isocyanates and achieves curing of the thermoset at room temperature, contrary to NIPU materials, which have to be cured at much higher temperatures [41,48]. 

**Figure 1:**
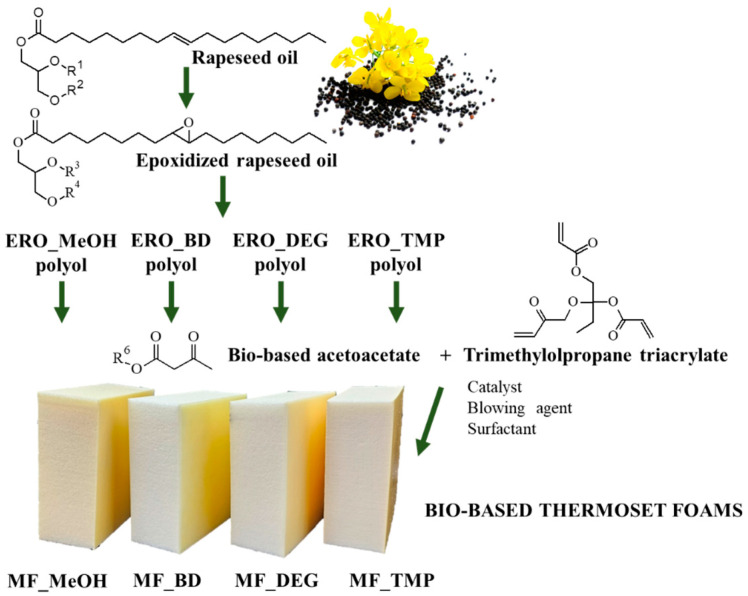
Bio-based thermoset foam development from RO.

This study used four different bio-based Michael donors to develop a thermoset polymer foam. The bio-based donors were obtained from industrial crop feedstock RO with varied functionality of acetoacetate (AA) groups. Similar to rigid PU foams, the functionality of the reacting components is one of the main factors that will influence the final material’s properties. By choosing high functionality components, the polymer matrix will be highly cross-linked, thus stiffer, and the material will have higher mechanical strength characteristics. On the contrary, a lower functionality component will yield a polymer matrix with lower crosslinking density and lower mechanical strength. Furthermore, the functionality of the reacting components will significantly influence the reactivity of the system as the higher functionality components will have a higher reactivity due to the higher content of the reacting groups present in the system. The schematic approach of this study is depicted in Figure 1.

A bio-based Michael donors with varied AA group functionality were used to develop a foamed thermoset polymer. The functionality was changed by changing the chemical structure of the bio-based polyols that are a precursor of the bio-based Michael donors. All four RO-based bio-polyols were obtained from ERO by epoxy ring opening reaction with varied alcohols. Following, epoxy ring opening agents were used, methanol (MeOH), butanediol (BD), diethylene glycol DEG and trimethylolpropane (TMP), and the OH group functionality of these alcohols is as follows 1, 2, 2 and 3, respectively. The characteristics of RO-based Michael donors and their synthesis procedure are described in detail in our previous work [49]. In the current study, we focus on foamed polymer development. 

### 3.1. Foaming Parameters

**Figure 2:**
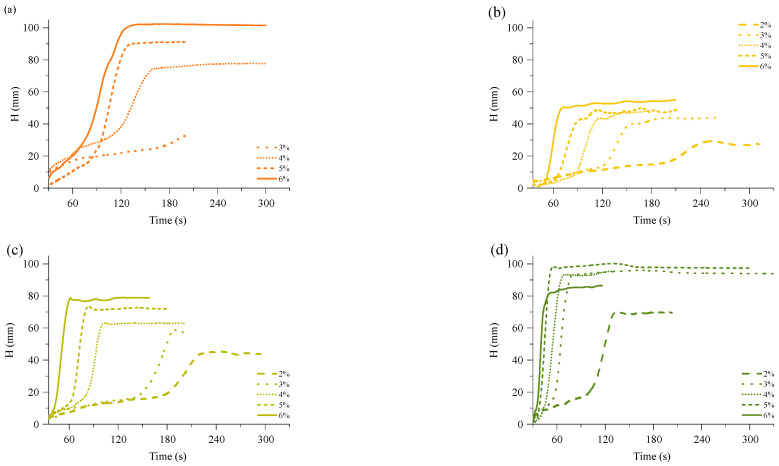
Foam height rise curves of RO-based Michael donor thermoset foams obtained from (**a**) ERO_MeOH_AA; (**b**) ERO_BD_AA; (**c**) ERO_DEG_AA and (**d**) ERO_TMP_AA.

The bio-based thermoset foams were obtained by mixing reacting components summarised in Table 1. The TMG catalyst content was optimised to select the proper catalyst content to obtain a foamed polymer at the laboratory scale. A foaming kinetics were recorded using FOAMAT®285 equipment which is equipped with an ultrasonic sensor that measures the change of the height of the material during the process. The foam raise curves are depicted in Figure 2. The TMG catalyst content was used in the following contents: 2%, 3%, 4%, 5%, and 6% of Michael donor mass, and it had a significant influence on the kinetic of the foam rise. As expected, the highest catalyst content delivered the fastest foaming of the material. Furthermore, the RO-based Michael donor with the highest functionality had the highest reactivity and vice versa. In the case of MF_MeOH foams with 2% of TMG catalyst, the foam did not cure due to the too low relativity of the Michael donor. The foaming of the material did not start as there was no heat generated from the curing reaction. The catalyst content had a direct influence on the maximum foam height, which increased with the increase of TMG catalyst content. In the case of ERO_TMP_AA based foam with 6% TMG catalyst content, the foam height was lower than for foams produced with TMG catalyst load of 3%, 4% and 5%. This was due to the fast reactivity of the 6% catalyst content foam. The foamed material solidified before it reached its maximum height. This is highly undesirable, as it leads to the formation of inner stresses in the material. Thus, an optimal catalyst content was selected for a smooth foaming process to balance the foaming and gelling processes”.

**Figure 3:**
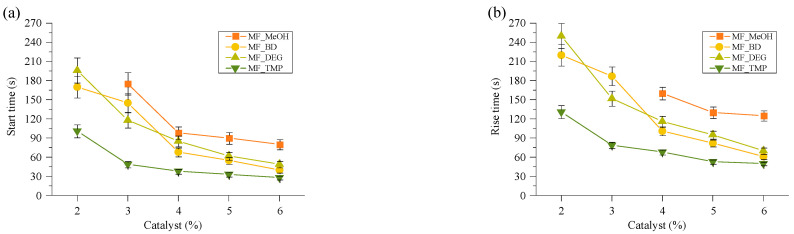
Bio-based thermoset foam kinetic parameters (**a**) foaming star time and (**b**) foam rise time.

The best summary of the catalyst influence on the foaming kinetics of the material can be seen in Figure 3, where the foaming start time and foam rise time are depicted. The foaming start time shows the start time of the foaming process and is characterised as the time when 5% of the final foam height has been reached. The foaming rise time was determined by the cross point between tangential lines after the foaming process and the tangential line of the foam rise curve. Both graphs elucidate that the foaming kinetics are related to the functionality of the used Michael donor. The MF_MeOH foams had the slowest foaming parameters, while MF_TMP were the fastest. Moreover, the MF-BD and MF-DEG foams’ foaming kinetics were relatively similar due to their similar chemical structure and reacting group functionality. The screening of the catalyst amount allowed the selection of the optimal TMG catalyst content for further experiments, which was as follows 6 pbw for MF_MeOH, 5 pbw for MF_BD and MF_DEG, and 4 pbw for MF_TPM. The selected catalyst content allowed us to obtain bio-based thermoset foams with a start time of 50–90 s and a rise time of 90–150 s. The catalyst content was selected due to convenience, as the foamed polymer was obtained at a laboratory scale in a steel mould. A faster reactivity would not allow to pour out the foam mass, and at a smaller catalyst amount, the foam did not cure fully.

**Figure 4:**
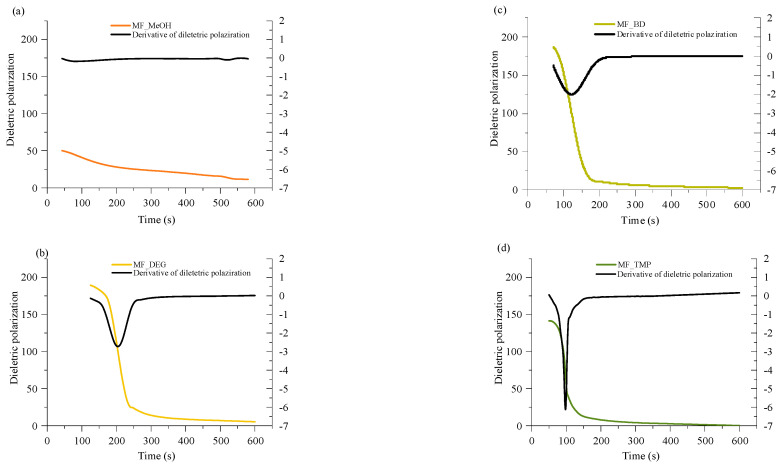
Dielectric polarization change and derivative of the dielectric polarization during the polymerization reaction of (**a**) MF_MeOH; (**b**), MF_BD; (**c**), MF_DEG; and (**d**), MF_TMP.

A convenient way to study thermoset curing is to measure the change of dielectric polarisation of the material during its reaction. The curves of dielectric polarization during the reaction are shown in Figure 4. During the polymerization reaction of thermosets, the dielectric polarization decreases, which is attributed to the formation of high molecular weight compounds as the components undergo polymerisation. As the oligomer/polymer chains grow and intermolecular interactions strengthen, the mobility of the polymer segments decreases, which restricts the ability of the oligomer/polymer chains to respond to an applied change of electric field, leading to a decrease in dielectric polarization. This change in dielectric properties during polymerization is crucial to consider in the design and development of thermosets for various applications tailored curing is needed, such as composites, foamed polymers and coatings.

**Figure 5:**
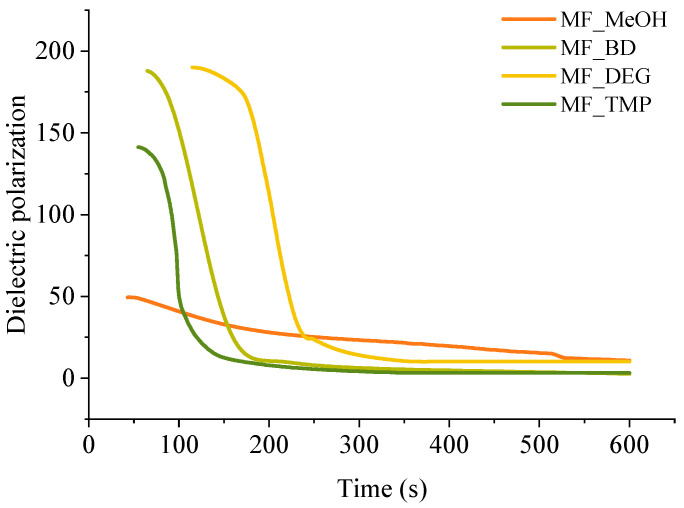
Dielectric polarization of the bio-based thermoset foams during the curing.

All RO-based Michael donors have relatively large molecular weights compared to TMPTA Michael acceptors, thus, the TMPTA has the largest influence on the dielectric polarisation measurement. As the Michael donor used for MF_MeOH has the lowest functionality, it requires the least amount of TMPTA, thus, the values of the dielectric polarisation are relatively low compared to other polymers. Furthermore, MF_MeOH material generates less heat during polymerization due to the low functionality of the Michael donor, thus explaining why data obtained from MF_MeOH has the least steep curve and MF_TMP—the steepest curve (see Figure 5).

When higher functionality acetoacetates were used (MF_TMP in Figure 5), the dielectric polarization decreased sooner. This can be attributed to the increased cross-linking and network formation that occurs with higher functionality acetoacetates during the polymerization process. As the functionality of used acetoacetate increases, the number of reactive sites available for cross-linking also increases, leading to a more rapid formation of oligomers. These cross-links and higher molecular weight compounds restrict the mobility of segments and limit the ability of the polymer chains to respond to the applied electric field. Consequently, the dielectric polarization drops sooner than when lower functionality acetoacetates are used (see MF_MeOH in Figure 5). The accelerated decrease in dielectric polarization with higher functionality acetoacetates highlights the influence of cross-linking density on the dielectric behaviour of polymeric materials. It accentuates the importance of understanding the impact of monomer functionality on the properties of polymers.

The dielectric polarization derivative curve typically exhibits a distinct peak and the midpoint of this peak corresponds to the gelling time for the polymer. When the dielectric polarization reaches values close to zero, the material achieves the desired level of cross-linking and molecular rearrangement, forming a strong and stable polymer network. The curing time for obtained polymeric materials is shown in Table 3. The curing time of developed bio-based polymers followed a similar relation to the foaming kinetics parameters, as the functionality of the Michael donor increasing, the curing time decreased. When a component has a lower functionality, such as in the case of MF_MeOH, it means that it has fewer available sites for cross-linking. As a result, it takes more time for these limited reactive sites to form the necessary bonds and create a three-dimensional network structure. Higher functionality components such as MF_TMP possess a greater number of reactive sites, which facilitates faster cross-linking and thus reduces the overall curing time. As the curing reaction is exothermic, the higher functionality components further accelerate the curing of the polymer due to the heating of the reacting mass. 

However, there was a noticeable discrepancy between MF_BD and MF_DEG thermoset foam samples. While the foaming start time of these samples was similar to 60 s at 5 pbw of catalyst, the midpoint of dielectric polarisation peak was almost two times sooner for MF_BD than for MF-DEG (120 s and 210 s, respectively). We attribute this discrepancy to the chemical structure of the Michael donors themself. The Michael donor used for production of MF_DEG has ester groups derived from DEG, while Michael donor used for production of MF_BD does not. The relatively polar moiety of ester oxygen might affect the dielectric polarisation sensor and could allow better mobility of the oligomer/polymer chains with the changing polarity of the electric field, thus prolonging the dielectric polarisation of the thermoset. Such a phenomenon was unexpected and warrants a further study of the chemical structure influence on the measurement of the thermoset curing using dielectric polarisation change over time.

The obtained results showed that it is possible to cure developed polymer at room temperature in a similar timeframe as rigid PU foam material [50]. The curing time for the developed bio-based polymers was 1–10 min. The rapid curing of the polymer matrix is necessary to obtain foamed polymer morphology, which will be done in further work packages.

### 3.2. Development of Bio-Based Thermoset Foams 

#### 3.2.1. Morphology of Developed RO-Based Thermoset Foams

**Figure 6:**
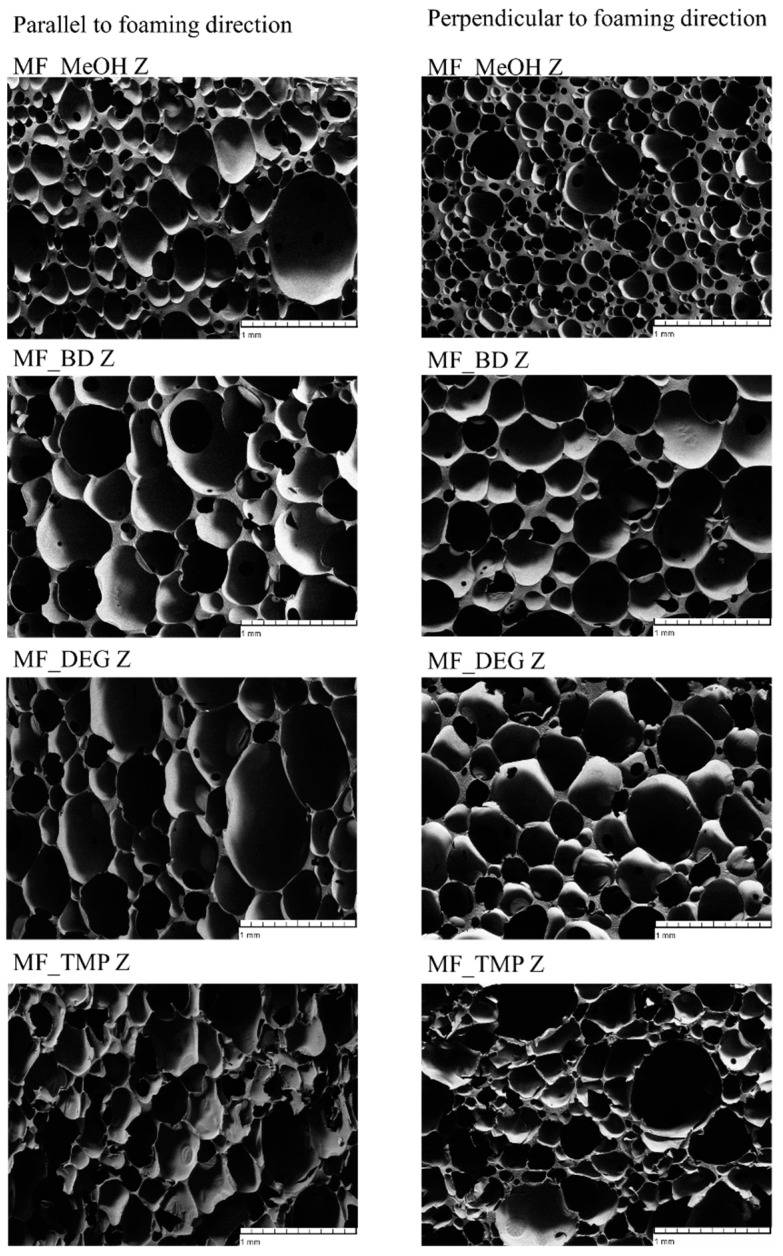
SEM images of RO-based thermoset foams parallel and perpendicular to the foaming direction.

The bio-based thermoset foams were obtained by mixing the Michael donor and acceptor components and pouring the mass of the reacting foam into a closed steel mould. Afterwards, the material was cured in the oven for 90 min to reach the final curing of the material. Obtained foams had even cell structure, which is depicted in Figure 6. There is slight anisotropy of the cells depending on the foaming direction. The cells are elongated in parallel to the foaming direction. The MF_MeOH has a smaller cell size compared to other RO-based Michael donor foams, which is explained by the low reactivity of the MF-MeOH donor. As the heat inside the reacting mass was generated more slowly, the nucleation of the physical blowing agent occurred much later. The slow foaming of the MF_MeOH foams was confirmed in Figure 3. As the formation of the foam bubbles happened later, when the polymer started to cure, the viscosity of the mass already was increased. The new bubbles were not able to merge, thus the small cell size of the MF_MeOH foam. The MF_BD and MF_DEG had typical cell sizes for polymer foams of 200–300 µm [51,52]. The MF_TMP had a different problem than MF_MeOH foams. The curing of the material happened faster than the foam rise (see Figure 3b and Figure 4d). Thus, the material wanted to expand when the polymer was already cross-linked. This led to the formation of inner stresses in the material, forming cracks inside the foam block. Such undesired effects might be solved by developing different catalyst systems with less rapid curing of the foam.

Moreover, the functionality of the MF_TMP might be too high to use this Michael donor as a pure component. In the rigid PU foam industry, a polyol component usually consists of several polyols. Similarly, the RO-based thermoset foams could be optimized by mixing components with different functionality. In the frame of this study, neat RO-based Michael donors were used to develop polymer foam and to compare their properties. 

#### 3.2.2. Common Characteristics of Developed RO-Based Thermoset Foams

The morphological characteristics, apparent density and thermal conductivity of the developed bio-based thermoset foams are summarized in Table 4. Unfortunately, the developed foams had very low closed cell content of 6–25%, which is not ideal for thermal insulation applications. The selected surfactant is designed for rigid PU foam application, and the chemistry of bio-based Michael addition thermoset foams is much different. A further investigation of different surfactants must be carried out to find a compound that delivers a closed-cell foam morphology. The low content of the closed cells means that the blowing agent is not trapped inside the foamed material, thus, the thermal conductivity of the bio-based foams depends on the thermal conductivity of air. The thermal conductivity of air is much greater than the thermal conductivity of Opteon™1100 (26.5 and 10.7 mW/(m·K), respectively) [53]. The apparent density of the developed bio-based thermoset foams was 140–225 kg/m^3,^ which can be considered acceptable, although it should be reduced for a commercial application of the material. The foamed material was considered almost fully cured, which was confirmed by FTIR analysis depicted in Figure S1. The MF_MeOH foam had a distinctive peak of unreacted acrylate groups at 1650 cm^−1^ due to insufficient temperature released during the foaming of the material. The thermal insulation coefficient of the developed RO-based thermoset foams ranged from 49.9 to 33.9 mW/(m·K), decreasing with the increase of the functionality of the Michael donor used for the foam preparation. The thermal conductivity of ~34 mW/(m·K) is acceptable for the thermal insulation materials. However, the thermal insulation properties may be further improved if a closed-cell morphology is achieved, which requires the development of new types of surfactants. 

### 3.3. Thermo-mechanical Properties of Obtained RO-Based Foams 

The thermo-mechanical properties are one of the most important parameters of the material, and they are the main factor deciding the application of a given material. The main parameters determining polymers exploitation are the thermal stability and the glass transition temperature, thus, this paragraph will explore the RO-based Michael donor influence on the thermos-mechanical properties of the developed foams. 

#### 3.3.1. TGA Results

**Figure 7:**
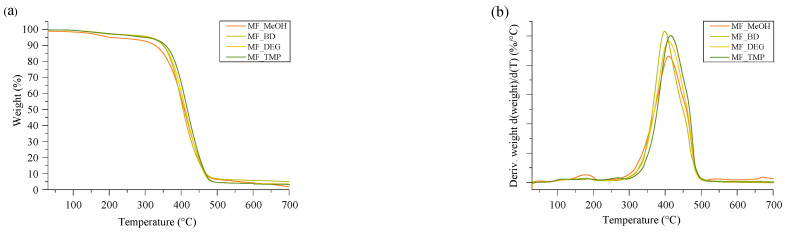
TGA curves of RO-based thermoset foams. (**a**) Is the mass loss curve and (**b**) is the derivative of the mass loss.

Thermal stability is the main parameter that characterizes applicability in engineering solutions requiring increased temperature. The developed RO-based thermoset foams had similar thermal stability, as seen in Figure 7. The mass loss occurred as one degradation step at a relatively high temperature of 396–414 °C. The mass loss derivative peak was relatively narrow and even without pronounced shoulders, which correlates to a homogeneous polymer material. The MF_MeOH had a small mass loss peak at 178 °C, which was attributed to unreacted small molecular components. The unreacted groups of the MF_MeOH foam polymer matrix were also confirmed using the FTIR spectra depicted in Figure S1. 

The summary of the thermal stability properties of RO-based thermoset foams is depicted in Table 5. The onset of the major mass loss was also relatively similar between the different foamed thermosets. The mass loss onset of MF_MeOH and MF_BD was almost the same at 360 °C, and the mass loss onset of MF_DEG was marginally higher at 363 °C. This could be explained by the structure of DEG. The oxygen introduced into the polymer matrix by DEG could form hydrogen bonding that could slightly increase the thermal stability of the polymer matrix in comparison to Michael donor ERO_BD_AA. Lastly, the MF_TMP foam had the highest mass loss onset temperature of 372 °C, which is explained by the increased cross-linking density of the polymer matrix due to the high functionality of the ERO_TMP_AA Michael donor. 

#### 3.3.2. The Glass Transition Temperature of RO-Based Thermoset Foams

**Figure 8:**
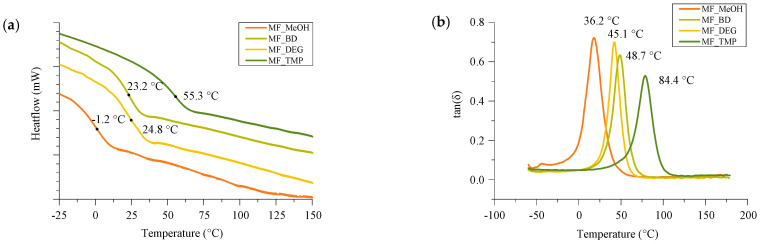
T_g_ analysis of developed RO-based thermoset foams. (**a**) Is the DSC and (**b**) is the tan(δ) curves.

The glass transition temperature (T_g_) of the developed RO-based thermoset foams was characterized using DSC and DMA methods (see Figure 8). Both analysis methods allow to determine the glass transition region of the polymer matrix. However, the studied phenomena are quite different; thus, the determined T_g_ can have discrepancies. The determined T_g_ values are summarized in Table 6. The determined T_g_ values were directly proportional to the crosslinking density of the polymer matrix, which is dependent on the functionality of the used bio-based Michael acceptor. The T_g_ of MF_MeOH foam was −1.2 °C. The material is not glassy at room temperature, which was also confirmed by the compression strength tests and by organoleptic methods. The developed MF_MeOH foam can be classified as flexible foam. Generally, the T_g_ in DMA analysis is a bit higher due to the mechanical glassification of the polymer. However, in the case of MF_MeOH there was a relatively high difference between the peak of tan(δ) in the first heating and cooling cycles 18.3 °C and 36.2 °C, respectively (see Figure 8b and Figure S2). This is related to the post-curing of the polymer material, which was activated by a change in the material’s thermal history. The tan(δ) peak change for the other three foams was not so significant, which entails a higher conversion of the reacting components. The other three foams can be classified as rigid foams as the tan(δ) peak of MF_BD; MF_DEG and MF_TMP foams were 48.7 °C, 45.1 °C and 84.4 °C, respectively.

The obtained results demonstrate that changing the functionality of the reacting RO-based Michael donors makes it possible to obtain a material with varied thermo-mechanical properties. The T_g_ can be designed for the specific application of the foam. The flexible RO-based thermoset foams have the potential for applications as a cushioning material in furniture and automotive industries or as an acoustic insulation material. Whereas the rigid glassy RO-based thermoset foams can be applied as thermal insulation. 

### 3.4. Compression Strength of Developed RO-Based Thermoset Foams

**Figure 9:**
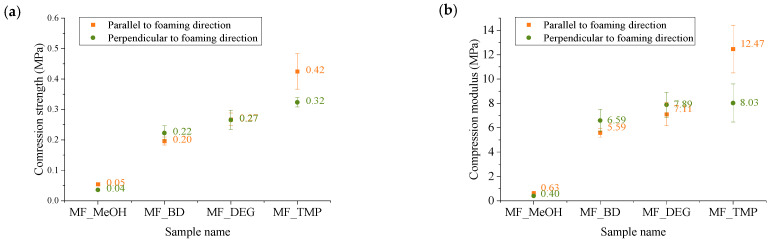
Mechanical properties of developed RO-based thermoset foams. (**a**) Is the compression strength and (**b**) is the compression modulus.

Compression strength is a mechanical property used to measure the ability of a material to withstand compressive (pushing or squeezing) forces without undergoing deformation or failure. a crucial parameter in materials science and engineering, as it helps determine how well a material can withstand loads that tend to compress or squash it. The compression strength of a material is typically represented as the maximum load or force that the material can withstand per unit area before it collapses or deforms. For rigid foam material, a compression strength at 10% of deformation is usually determined, or the maximum compression strength before the plastic deformation plateau. Determined compression strength and compression modulus of elasticity are depicted in Figure 9. 

**Figure 10:**
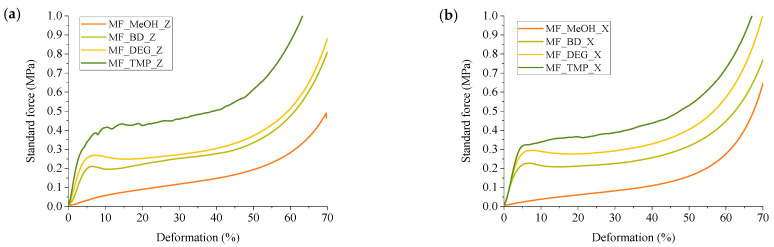
Stress-stain curves of developed RO-based thermoset foams. (**a**) Is the parallel to the foaming direction and (**b**) is the perpendicular to the foaming direction.

The functionality of the RO-based Michael donor had a distinctive influence on the compression properties of the developed thermoset foams. The MF_MeOH foams had the lowest compression strength and compression modulus. The MF_MeOH foam T_g_ was below room temperature, so the material was deformed as a viscoelastic foam. The stress–strain curves are depicted in Figure 10. The plastic deformation region can be seen until ~50% deformation, after which the densification of the foam occurs. Unfortunately, the hysteresis experiments have not been performed for the MF_MeOH foam, but the presented data shows that the developed foam can be used as a flexible foam material. The MF_MeOH foam has a slight anisotropy where the compression strength and compression modulus are slightly higher parallel to the foaming direction, as seen in Figure 9 due to cell elongation in the foaming direction. 

The MF_BD and MF_DEG foams both have very similar mechanical properties as both Michael donors had similar functionality; thus, a similar cross-linking density was achieved in the polymer matrix. The foams were isotropic in the margin of error, and the cell size anisotropy did not influence the mechanical properties. The MF_DEG foams had slightly higher compassion strength and compression modulus when compared to MF_BD. This difference can be explained by the MF_DEG foam having a slightly higher apparent density of 166 kg/m^3^ to 147 kg/m^3^ in the case of MF_BD. Furthermore, the polymer matrix of MF_DEG foam is stiffer due to hydrogen bonding that occurs with the oxygen atom of the DEG moiety. The stress–strain curves of MF_BD and MF_DEG (depicted in Figure 10) have a close resemblance to a typical rigid PU foam compression stress–strain curve. However, the mechanical properties are relatively low for such a high apparent density. The typical PU foam contains plenty of rigid segments in its chemical structure derived from the hydrogen bonding of urethane, polyurea and isocyanurate groups [54]. Furthermore, rigid PU foams have a stiff aromatic group derived from the aromatic pMDI used in its production. The developed RO-based thermoset foams lack aromatic groups; thus, the obtained compression properties can be considered a success as the compression strength of 0.21 MPa is enough to be used as a thermal insulation material. 

Lastly, the MF_TMP foam had the highest compression strength and compression modulus in parallel to the foaming direction, 0.42 MPa and 12.5 MPa, respectively, due to the highest functionality of the RO-based Michael donor. Unfortunately, it was difficult to obtain a good quality MF_TMP sample due to crack formation during the foaming process. This could be solved by changing the catalyst system or by mixing different Michael donors in the foam formulations. The microcrack formation in the foam material can also be seen in the rather large standard deviation of the samples in Figure 9. The foam samples visually had no defects, but during the compression test, the microcracks formed during the foaming process decreased the mechanical properties. 

## 4. Conclusions

An industrial crop product—rapeseed oil was used to develop bio-based thermoset foams via Michael addition reaction. Obtained foams had varied physico-mechanical characteristics ranging from flexible to rigid foams. The proposed process is a direct alternative to polyurethane foam production technology as the foamed thermoset is obtained by mixing two liquid components that cure at room temperature. This approach allows for a bypass of the use of isocyanates which is one of the significant drawbacks of polyurethane materials. By changing the functionality of the bio-based Michael donors it was possible to obtain foams with different properties and glass transition temperatures ranging from −1.2 to 55.3 °C. The MF_MeOH foams were characterized as flexible foams whereas the other three materials were rigid thermoset foams. The potential application of MF_MeOH is as a cushioning material in the automotive and furniture industry or as acoustic insulation in civil engineering and appliance production. The other three foams MF_BD; MF_DEG and MF_TMP can be applied as thermal insulation in civil engineering due to the relatively low thermal conductivity of 33.9–35.4 mW/(m·K).

## Figures and Tables

**Table 1:** Formulations of the Michael foams.

Components		MF_MeOH	MF_BD	MF_DEG	MF_TMP
				Pbw	
**Michael donor**	MeOH_AA	100	-	-	-
	BD_AA	-	100	-	-
	DEG_AA	-	-	100	-
	TMP_AA	-	-	-	100
**Michael acceptor**	TMPTA	47.6	77.2	77.1	85.9
**Catalyst**	TMG	2.0–6.0	2.0–6.0	2.0–6.0	2.0–6.0
**Surfactant**	TG_B	5.0	5.0	5.0	5.0
**Blowing agents**	Solstice^®^ LBA	35	35	35	35

**Table 2:** Formulation of the developed polymeric materials.

Acronym	Michael Donor	Michael Acceptor	Catalyst
		Pbw	
MF_MeOH	7.04	2.96	0.21
MF_BD	5.64	4.36	0.17
MF_DEG	5.65	4.35	0.17
MF_TMP	5.38	4.62	0.16

**Table 3:** The curing times for different functionality bio-based thermoset foams.

Polymeric Material	Curing Time Intervals, s	The Midpoint of Peak, s
MF_MeOH	475–550	525
MF_BD	60–200	120
MF_DEG	160–250	210
MF_TMP	50–125	95
PU curing [50]	23–50	23–49

**Table 4:** Summary of morphology parameters and typical characteristics of the developed Michael foam.

	MF_MeOH	MF_BD	MF_DEG	MF_TMP
Closed cell content, vol.%	24.73 ± 0.19	5.90 ± 0.12	5.73 ± 0.16	12.39 ± 0.22
Apparent density, kg/m^3^	225.56	147.23	166.29	139.78
Thermal conductivity ± 0.02, mW/(m·K)	49.89	33.96	35.35	33.89

**Table 5:** TGA analysis of RO-based Michael addition thermoset foam.

Sample	First Onset, °C		T_m5%,_ °C		T_m10%,_ °C		T_m25%,_ °C		T_m50%,_ °C		T Peak 1, °C		T Peak 2, °C	
MF_MeOH	359.9	±1.6	229.1	±24.9	332.9	±5.9	376.1	±2.6	409.1	±0.4	177.8	±1.3	408.7	±2.0
MF_BD	359.1	±1.5	308.2	±4.8	348.1	±2.4	377.2	±1.4	403.0	±0.9	-	-	395.9	±0.9
MF_DEG	362.6	±0.4	313.9	±0.9	348.9	±0.6	382.0	±0.9	410.8	±1.7	-	-	408.6	±2.1
MF_TMP	371.5	±1.3	298.8	±2.0	354.5	±0.5	388.4	±0.8	415.5	±1.0	-	-	414.3	±1.6

**Table 6:** The glass transition temperature of RO-based Michael addition thermoset foam.

Sample	Glass Transition Temperature (°C)		
	T_g(DSC)_	T_g(DMA)_ Heating	T_g(DMA)_ Cooling
MF_MeOH	−1.17	18.27	36.20
MF_BD	23.20	49.07	48.66
MF_DEG	24.83	42.47	45.05
MF_TMP	55.29	78.85	84.41

## Data Availability

The authors declare that all data supporting the findings of this study are available within the article. Extra data are available from the corresponding author upon request.

## Supplementary Materials

The following supporting information can be downloaded at: https://www.mdpi.com/article/10.3390/polym16010117/s1, Figure S1: FTIR spectra of RO-based thermoset foams; Figure S2: tan(δ) of the cooling cycle of DMA analysis for RO-based thermoset foams.
