# Supplementary material for: Rapeseed Oil as Feedstock for Bio-Based Thermoset Foams Obtained via Michael Addition Reaction
Source: Polymers (Basel). 2023 Dec 29;16(1):117. doi: 10.3390/polym16010117 (PMC10780781; doi:10.3390/polym16010117)
Supplement: Supplementary file 1 [file polymers-16-00117-s001.zip › polymers-2768567-supplementary.pdf]

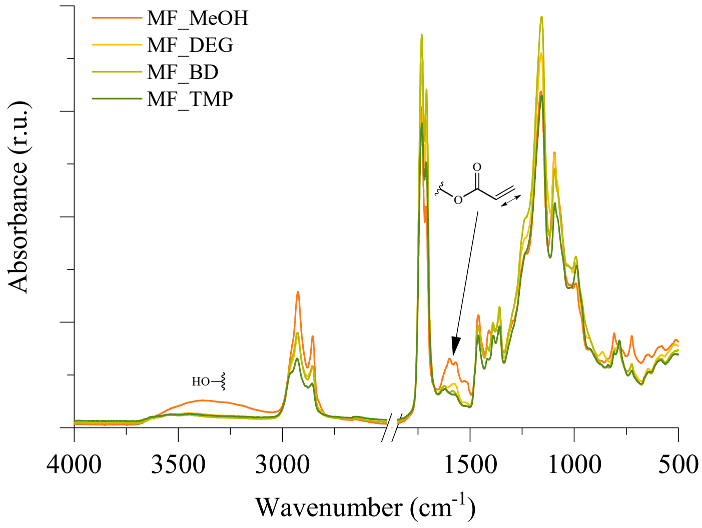

Figure S1. FTIR spectra of RO-based thermoset foams

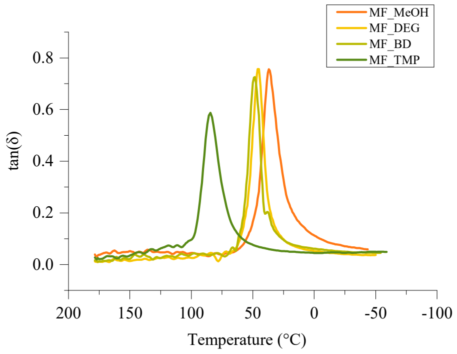

Figure S2. tan(δ) of the cooling cycle of DMA analysis for RO-based thermoset foams
